# Characterization of Two Satellite DNA Families in the Genome of the Oomycete Plant Pathogen *Phytophthora parasitica*

**DOI:** 10.3389/fgene.2020.00557

**Published:** 2020-06-05

**Authors:** Franck Panabières, Corinne Rancurel, Martine da Rocha, Marie-Line Kuhn

**Affiliations:** INRAE, UCA, CNRS, ISA Sophia Antipolis, Biot, France

**Keywords:** satellite DNA, genome structure and organization, *Phytophthora*, evolution, centromere, telomere

## Abstract

Satellite DNA is a class of repetitive sequences that are organized in long arrays of tandemly repeated units in most eukaryotes. Long considered as selfish DNA, satellite sequences are now proposed to contribute to genome integrity. Despite their potential impact on the architecture and evolution of the genome, satellite DNAs have not been investigated in oomycetes due to the paucity of genomic data and the difficulty of assembling highly conserved satellite arrays. Yet gaining knowledge on the structure and evolution of genomes of oomycete pathogens is crucial to understanding the mechanisms underlying adaptation to their environment and to proposing efficient disease control strategies. A *de novo* assembly of the genome of *Phytophthora parasitica*, an important oomycete plant pathogen, led to the identification of several families of tandemly repeated sequences varying in size, copy number, and sequence conservation. Among them, two abundant families, designated as *PpSat1* and *PpSat2*, displayed typical features of satellite DNA and were collectively designated as *PpSat*. These two satellite families differ by their length, sequence, organization, genomic environment, and evolutionary dynamics. *PpSat1*, but not *PpSat2*, presented homologs among oomycetes. This observation, as well as the characterization of transcripts of *PpSat* families, suggested that these satellite DNA families likely play a conserved role within this important group of pathogens.

## Introduction

Most eukaryotic genomes are mainly composed of repetitive sequences which may take into account a main proportion of total nuclear DNAs (Charlesworth et al., 1994; Haas et al., 2009; Lee and Kim, 2014). These sequences generally include multigene families, as well as two major groups that are classified according to their abundance and genomic organization: tandem repeats, including micro-, mini, and satellite DNA (SatDNA), and transposable elements (TEs), which are, by definition, mobile and tend to spread across the genome (Padeken et al., 2015). The abundance and diversity of both SatDNA and TEs may vary from one organism to another within a given species or genus (Razali et al., 2019; Louzada et al., 2020). TEs are further divided into two main classes, DNA transposons, which transpose via a cut-and-paste mechanism, and retrotransposons, which transpose via a copy-and-paste mechanism, and they have a huge expansion potential and constitute an important (up to 70%) fraction of the genome (Wicker et al., 2007). SatDNA families generally consist of 150–500-bp head-to-tail tandemly repeated sequences (Garrido-Ramos, 2017), present in hundreds to thousands of copies, and compose a main constituent of heterochromatin, often found at centromeric, pericentromeric, and telomeric regions (Tek and Jiang, 2004; Garrido-Ramos, 2017; Hartley and O’Neill, 2019). Centromeres constitute the region at which spindle microtubules attach to the kinetochores to ensure the faithful segregation of chromosomes during cell division (Henikoff and Dalal, 2005). They thus play a pivotal role in cellular homeostasis. Satellite DNA families may also be found in interspersed locations (Brajković et al., 2018). In addition, centromeres not only host SatDNA but also are highly repeated centromere-specific retrotransposons (Presting, 2018). SatDNA has long been considered as junk or selfish DNA, as no function could be attributed to this genomic complement and because it was considered to persist over generations at the expense of the rest of the genomes (Doolittle and Sapienza, 1980; Orgel and Crick, 1980). SatDNA is now, like TEs, considered as an important driver of genome architecture and plasticity, as well as evolution and speciation (Presting, 2018; Louzada et al., 2020).

Repetitive DNA may also contribute to virulence of pathogenic microbes, as proposed in the case of filamentous plant pathogens, among which are fungi and oomycetes (Razali et al., 2019). These eukaryotic organisms are phylogenetically distant (Adl et al., 2019), but they share several features such as filamentous growth and common ecological niches and hosts. Common infection strategies have also evolved in these organisms, such as stage-specific infection structures and the ability to deliver into plant cells hundreds of effectors, which are generally small proteins that manipulate or defeat defense mechanisms and divert plant functions for successful infection (Franceschetti et al., 2017). The availability of complete sequences from both fungi and oomycetes further revealed additional commonalities, one of the most intriguing being the modular architecture of their genomes. Hence, microbial genomes are composed of gene-rich regions alternating with gene sparse compartments enriched in repetitive sequences and TEs, leading to the emergence of the “two-speed genome” concept for filamentous pathogens (Raffaele and Kamoun, 2012). More surprisingly, a large amount of effector genes was found to be associated with these gene sparse regions in a number of genomes (Haas et al., 2009; Dong et al., 2015). Hence, the impact of the genomic environment plasticity on the virulence in host organisms may be associated with the relation between effector gene evolution and repetitive DNA sequences. The regulation of effector gene expression may also be under the influence of the global TE-driven translocations and chromosomal rearrangements (Haas et al., 2009; Grandaubert et al., 2014; Dutheil et al., 2016). Several reports validated this hypothesis (Kasuga et al., 2012), along with the evidence that a fungal effector may itself derive from a TE (Amselem et al., 2015). So, good knowledge on the landscape of repetitive DNAs of filamentous pathogens is crucial for a better understanding on their potential impact on the evolution and host adaptation of their host genomes. Despite the numerous commonalities evoked above, oomycetes, which are grouped with diatoms and brown algae within stramenopiles (Adl et al., 2019), differ from fungi by a number of features, such as metabolic and structural particularities, as well as a probable, although largely understudied, diploidy (Kamoun et al., 2015). The assembly of genome sequences allowed identifying a large catalog of TEs of oomycetes (Tyler et al., 2006; Haas et al., 2009; Baxter et al., 2010; Jiang et al., 2013; Liu et al., 2016; Ye et al., 2016; Ali et al., 2017; Yang et al., 2018; Malar et al., 2019), but it did not provide any information on their chromosomal organization. The ploidy level and the chromosome number have been determined in a handful of *Phytophthora* species (Sansome and Brasier, 1974; Sansome et al., 1975; Brasier et al., 1999), which are far from reflecting the diversity of the thousands of oomycete species, and variability and abnormalities appear to be common (Biasi et al., 2016). In addition, oomycete centromere locations are unknown and a preliminary analysis of telomeric regions has been reported in *Phytophthora infestans*, indicating a very high rate of intraspecific polymorphism (Pipe and Shaw, 1997). Moreover, no information is available on the diversity of satellite DNA in these organisms. The most likely reason is that these sequences, because of their repetitive nature and size, in which they are longer than the average read length generated by next-generation sequence (NGS) technologies (Thomma et al., 2016), as well as their extreme conservation triggered by concerted evolution (Dover et al., 1982) escaped to identification and were collapsed in most genome assemblies.

Aiming to gain insights on the molecular bases of oomycete virulence, we developed a study on *P. parasitica*, a soilborne pathogen that attacks up to 200 plant species worldwide (Panabières et al., 2016). *P. parasitica* as a species has a very broad host range, but many isolates belong to host-specific lineages (Colas et al., 1998; Biasi et al., 2016; Chowpadda et al., 2016) while some strains can attack a very large array of hosts, including the model plant species *Arabidopsis thaliana* (Panabières et al., 2016). This species therefore offers a unique opportunity to investigate on a genome-wide basis the relationships between mechanisms underlying general pathogenicity and those that govern host specificity. These would include variability in the effector repertoires, as well as the involvement of repeats and TEs in chromosomal shuffling and rearrangements. On the course of an international initiative, we generated a draft genome, based on Illumina sequencing, of PP INRA-310, a polyphagous strain initially collected on tobacco (Attard et al., 2008). A 111X coverage led to an assembly of ∼82.39 Mb, in accordance with the estimated size of the *P. parasitica* genome (Shan and Hardham, 2004). Yet, the total length of ungapped sequences only reached 53.87 Mb, suggesting that a substantial part of the genome was refractory to the current assembly. We thus intended to generate an improved version of this genome using PacBio technology. On the course of this sequencing effort, we identified several classes of repetitive sequences, among which are two families that displayed features of a satellite DNA but that dramatically differ by their organization, evolutionary dynamics, conservation among oomycetes, and transcriptional profiles. We report here the detailed analysis of these families, which constitutes to our knowledge the first description of oomycete satellite DNA families.

## Materials and Methods

### Biological Materials and Genome Sequencing

*Phytophthora parasitica* strain PPINRA-310 was maintained at the INRA ISA collection and grown on a V8 medium at 24°C for 10 days prior to extraction of genomic DNA as described (Panabières and le Berre, 1999). Library preparation and sequencing were performed at the GeT-PlaGe core facility, INRAE Toulouse. Assembly was performed using Falcon-Unzip v.0.7.3.

### Bioinformatic Analysis

Repeated sequences were identified with Tandem Repeats Finder (Benson, 1999) using default parameters. The exact start, end, and length of repeat units were assessed from examination of the different loci harboring the satellite arrays. The monomeric consensus sequences were used as queries in Blastn searches against the entire *P. parasitica* INRA 310 genome, then against the various oomycete genomes available at Ensembl and GenBank. GC content was calculated using the GC content calculator online^1^, with a window size ranging from 10 to 50 nucleotides. Pairwise comparisons were performed between the consensus and the variant sequences using Blastn at the NCBI using default parameters. Searches for known repetitive sequences and transposable elements were done at Repbase using the Censor tool (Bao et al., 2015). An initial assessment of internal repeats was performed manually. Potential secondary structures were investigated using RNAfold 2.4.13 (Gruber et al., 2008) implemented at the ViennaRNA Web server^2^. Structures displaying the lowest free energy values were selected, reflecting the most stable folding. Multiple alignments were conducted using Muscle (Edgar, 2004) implemented in SeaView 4.0 (Gouy et al., 2010). Phylogenetic trees were constructed by maximum likelihood (ML) using Mega7 (Kumar et al., 2016) using the best-fitting substitution model, which is the Tamura 3-parameter, with uniform rates of substitutions and 500 bootstrap replicates. A discrete gamma (+G) distribution was used to model evolutionary rate differences among sites (5 categories). Trees were drawn using the Interactive Tree Of Life (ITOL) online tool (Letunic and Bork, 2016). A phylogenetic network of the *PpSat families* was conducted using the SplitsTree software with the Neighbor-Net and uncorrected p-distance parameters (Huson and Bryant, 2006).

*PpSat1* and *PpSat2* expression was evaluated through evaluation of the total amount of reads matching satellite sequences in Blastn searches against RNA-Seq data available at GenBank (accessions SRX1124837–SRX1124840, SRX1124842–SRX1124845, SRX1124847–SRX1124868, SRX2727839–SRX2727852, and SRX4902085–SRX4902107).

## Results

### High Diversity of Tandem Repeat Families in *P. parasitica* INRA-310 Includes SatDNA Repeats

We generated a 115× sequencing coverage of the genome of *P. parasitica* PPINRA-310 using the PacBio sequence technology. This provided 10.1 Gb of long (N50 ∼ 15 kb) reads that were further assembled into 339 primary contigs, ranging from 20.20 to 2,685 kb (N50 ∼ 498 kb). This resulted into a 77.3-Mb genome, therefore uncovering >23 Mb of sequences awaiting annotation. On the course of *the* assembly, we identified 6 highly similar contigs of 20–25-kb length that were absent from the initial genome version. The GC content of these contigs was low (>37%), greatly deviating from the known average GC content observed in various *Phytophthora* genomes and contrasting our estimation of the overall *P. parasitica* GC content which is ∼50.2%. In addition, no significant ORF could be predicted from these sequences. Each scaffold consisted of 66–76 repeats of ∼330 bp, tandemly arranged in head-to-tail orientation. The length and nucleotide composition as well as the repetitive nature of the sequences constituting these scaffolds were characteristic of satellite DNA, which prompted us to evaluate the relative proportion of satellite DNA in the new *P. parasitica* assembly. We thus searched tandemly repeated sequences with a monomer size ranging from 100 to 500 bp, which is the common range for most satellite DNA families and present in the assembly in ≥100 copies. Analysis using Tandem Repeats Finder (TRF) revealed numerous consensus patterns that were sorted according to their size and base composition. Yet, pairwise comparisons of these sequences allowed retrieving only four distinct families obeying to our criteria, which were provisionally designated as *PpSat1* to *PpSat4*, according to their decreasing abundance (Table 1). Each sequence family was present in moderate to low copy number, the putative satellite family initially identified being the most abundant. Comparison of the consensus sequences of each family against Repbase revealed that *PpSat3* derived from retrotransposons of the Gypsy lineage, whereas *PpSat4* displayed the signature of a DNA transposon of the MuDR type (Table 1). *PpSat1* and *PpSat2* did not match to any repeat consensus hosted at Repbase. Further searches in GenBank assembly did not reveal significant matches so that these two families were considered as novel sequences.

**TABLE 1:** Characteristics for the most abundant tandem repeat families of *P. parasitica.*

		**Total**		**Longest**	**GC**			**% length**
**Sat**	**Length**	**Repeat**	**Scaffolds**	**Array**	**Content (%)**	**TE origin**	**TE**	**matching to TE**
*PpSat1*	337	513	18	>76	36.79	N	n	n
*PpSat2*	143	440	11	78	58.77	N	n	n
*PpSat3*	294	191	2	136	62.24	Y	Gypsy	88.09
*PpSat4*	420	150	2	82	65.23	Y	MuDR	63709

**n, not relevant.**

The consensus sequences of *PpSat1* and *PpSat2* generated by TRF were used to identify homologs and possibly dispersed copies in the genome. *PpSat1* was retrieved in 12 additional scaffolds, which harbored 1–34 repeats of 290–338 bp, as well as truncated repeats located at the boundaries of some arrays (Table 2). We then identified five scaffolds of 29.5–2186 kb that possessed a single unit varying from 326 to 337 bp, three scaffolds (445–1715 kb) yielding 6–10 repeats, and two scaffolds of ∼26 and ∼15 kb that possessed 22 and 34 repeats (Table 2). Last, two scaffolds possessed 2 and 11 repeats that were located at one end of the sequence, so it was not possible to estimate the extent of the array. Discarding truncated copies led to the generation of a 504-repeat dataset consisting of single-copy (SC, 5 repeats), low-copy (LC, 24 repeats), and high-copy (HC, 463 repeats) arrays, while 12 entire monomers constituted an ambiguous group (Table 2).

**TABLE 2:** Main features of the *P. parasitica PpSat* families.

		**Scaffold**			**Monomer**	
**Category**	**Scaffold**	**Length (bp)**	**Begin**	**End**	**In scaffold**	
***PpSat1***						
*Single copy*						
	P003	2186328	2185544	2185869	1	
	P051	591673	590881	591207	1	
	P087	181736	368	690	1	
	P135	91126	38070	38406	1	
	H312	29469	28766	29091	1	
*Low copy*						
	P007	1716993	1682615	1687068	10*	*1624 bp interrupting the array
	P046	586020	533491	534491	8*	*1213 bp interrupting the array
	11040	444249	381813	384992	6*	*1225 bp interrupting the array
*High copy*						
	P226	25323	1	25323	76	
	P231	23831	1	23831	72	
	P234	23083	1	23083	70	
	P236	22877	1	22877	70	
	P233	23110	1	23110	69	
	P244	21421	1	21421	66	
	H542	14558	3161	14588	34	
	P224	25855	18502	25855	22	
*Ambiguous*	H469	19290	15618	19290	11	
	H446	20511	19937	20511	2	
***PpSat2***						
*High copy*	P055	474503	460183	474010*	78	*2736 bp interrupting the array
	H357	26455	23053	23277	72	
	H033-1	502643	493142	500897	55	
	HI69	47928	40492	47928	53	
	P035	661506	651764	661024*	45	*2736 bp interrupting the array
	P044	614947	242	5446	38	
	H118	65056	59914	65056	36	
	P010	1478652	1339010	1345364*	26	*2761 bp interrupting the array
	HO10	1341877	1189115	1194049*	18	*2766 bp interrupting the array
*Ambiguous*	H059	272380	271581	272380	11	
	H033-2	502643	1	1033	8	

The situation was simpler in the case of *PpSat2*, which was organized only in high copy arrays (Table 2). As already observed with *PpSat1*, two scaffolds contained a moderate number of repeats, but the arrays were located at one end of the sequences. Discarding truncated copies revealed a 426-member family with a mean monomer length of 142 bp and a mean GC content of 57.43%.

**FIGURE 1:**
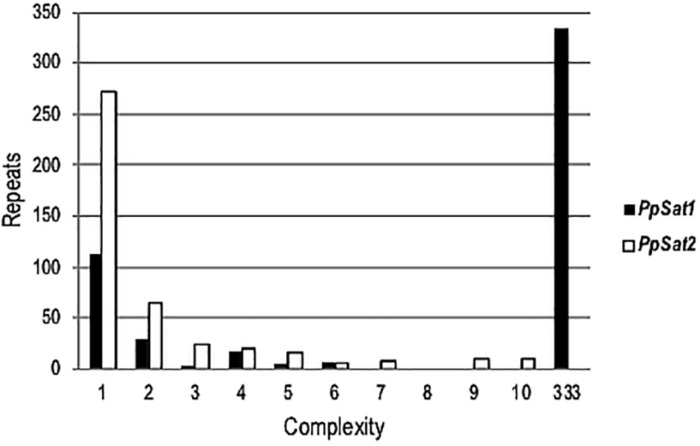
Complexity of *PpSat* sequences, illustrated by the distribution of the repeats into complexity classes where repetition level 1 = single copy, 2 = 2 identical copies, 3 = 3 identical copies, and so on.

To estimate the level of conservation of satellite families, pairwise comparisons were performed which revealed a total of 135 and 323 distinct sequences within *PpSat1* and *PpSat2*, respectively. Yet, the sequences were not equally represented. Hence, *PpSat1* complexity presented a bimodal distribution, as a monomer was present in 333 copies, while 113 sequences were unique, and 21 sequences were present in 2–6 copies. Conversely, *PpSat2* was mainly made of unique or low-copy sequences (Figure 1).

### *PpSat1* and *PpSat2* Define Two Satellite DNA Families With Different Dynamics

Despite an apparent diversity, *PpSat* sequences are highly conserved, as the most divergent monomers display 91.2 and 89.26% identity with the *PpSat1* and *PpSat2* consensus, respectively.

**FIGURE 2:**
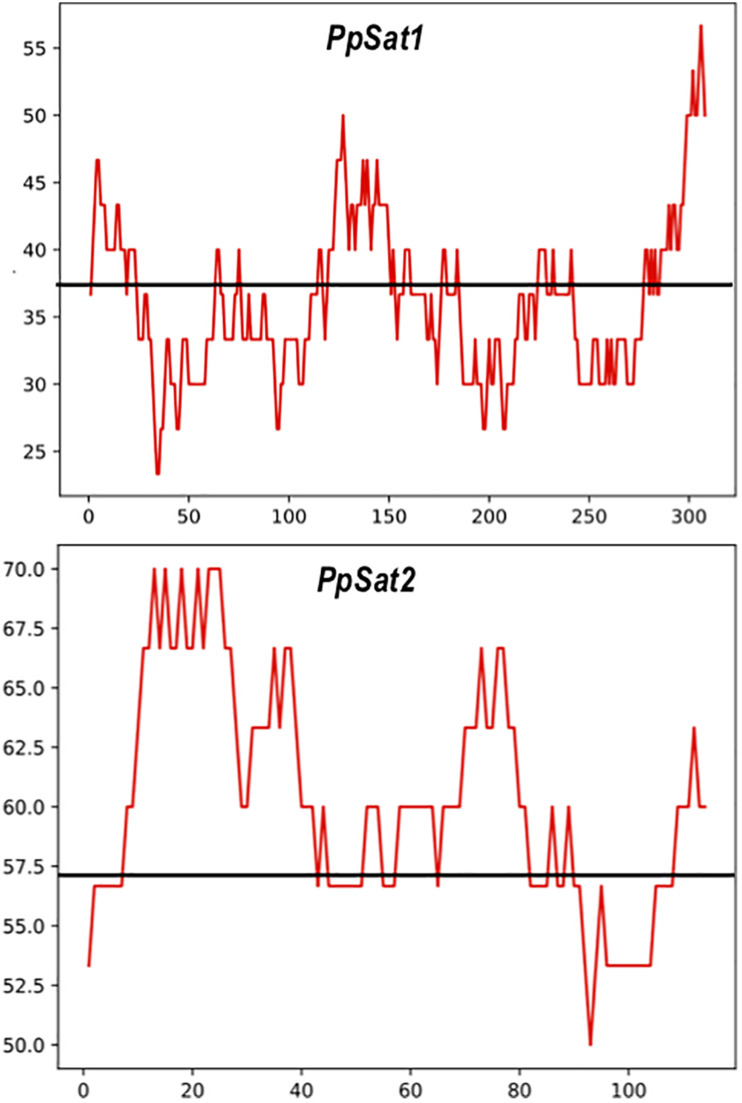
Distribution of the GC content across the *PpSat* consensus, as evaluated by sliding window analysis. The GC content presented in the figure was calculated using a sliding window of 30 nucleotides. The average GC content is shown by a solid line.

As a consequence of sequence conservation, the base composition was also highly conserved between repeats of the satellite families. The base distribution varied across *PpSat1* and *PpSat2*, revealing GC-rich and GC-poor segments deviating from the average GC content (Figure 2). A further analysis indicated that *PpSat2* was also AC rich (62.93%), especially in the central region of the monomer, and that the overall GC richness resulted from a rather relative paucity in T residues (13.28%).

**FIGURE 3:**
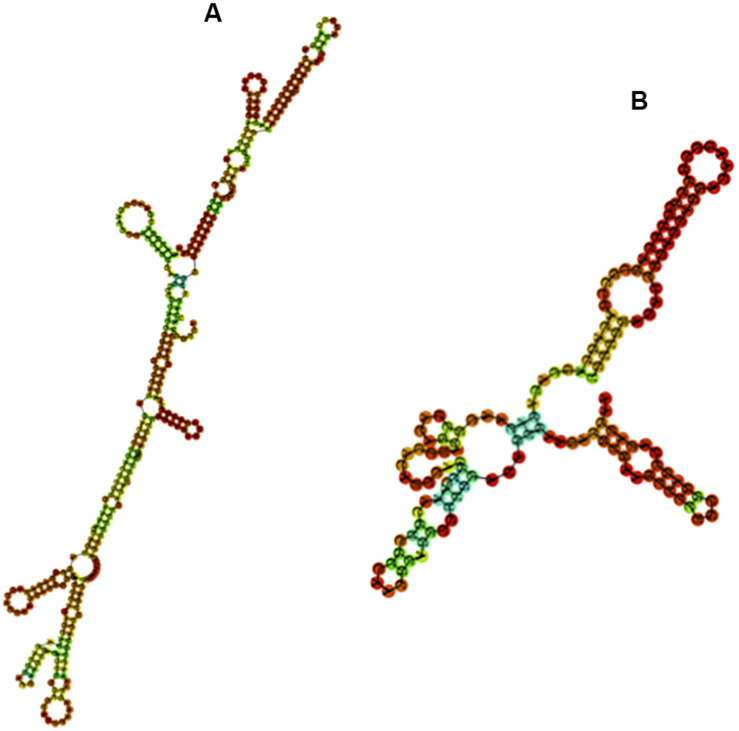
Predicted secondary structures of *PpSat* consensus. The most stable predicted secondary structures for *PpSat1* **(A)** and *PpSat2* **(B)** were generated at the ViennaRNA web server.

We further examined whether the base composition reflected a further structuration of the *PpSat* monomers into shorter motifs. *PpSat1* contained several homopolymeric (A_3__–__5_ and T_3__–__5_) stretches throughout the sequence, as expected from the overall high AT content. No such stretches were observed in *PpSat2*, which rather displayed (CA) repeats, as a consequence of its biased nucleotide composition. Despite the presence of several short direct repeats, we could not find traces of an internal repeated structure, so that the *PpSat* families did not arise from amplification of shorter satellites. We then aimed at determining the extent of secondary structure formation potential from single-strand *PpSat* DNA or transcripts, if these sequences are expressed. Results are presented in Figure 3. The free energy of formation of secondary structures was −82.66 and −38.4 kcal/mol for *PpSat1* and *PpSat2*, respectively, and complex secondary structures could be predicted (Figure 3). So, we could conclude that *PpSat1* and *PpSat2* may theoretically adopt stable secondary structures either as single-strand DNA or if transcribed.

### *PpSat1* and *PpSat2* Have Complex and Distinct Evolutionary Trajectories

**FIGURE 4:**
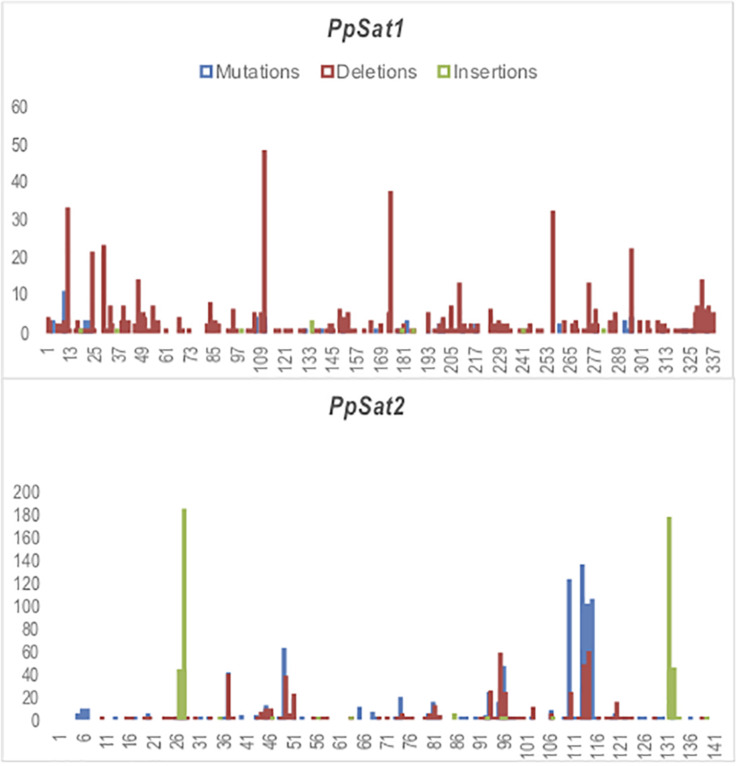
Identification of variable sites across *PpSat* repeats. The *x*-axis represents the nucleotide position and the *y*-axis the frequency of base substitutions (blue), deletions (red), and insertions (green) revealed by alignment with each consensus sequence.

We scored 682 and 1742 mutations (substitutions and indels) among *PpSat1* and *PpSat2* monomers, respectively. Considering that the length of the *PpSat1* monomers is more than two-fold the length of *PpSat2* and that they are more abundant, we could assume that *PpSat1* is less diverse than *PpSat2*. Substitutions took into account 46.72% of the total mutations observed in *PpSat2*, whether they represented only 9.53% of the mutations occurring within *PpSat1*. By contrast, deletions constitute 89% of the mutations in *PpSat1*, compared to 26.75% in *PpSat2*. We also noted that mutations were not randomly distributed across the monomers but rather accumulated in a few sites in each family (Figure 4). Analysis of *PpSat2* sequences further revealed that substitutions preferentially occurred in four regions where the local GC content is lower than the average, whereas indels were generally located in regions that outcompete the mean GC content. To further assess the consequences of these mutations on the overall *PpSat2* sequences, we examined the nature of the substitutions and indels. Substitutions induced a net rise in the GC content of *PpSat2*, although 31% of them were neutral. Deletions also led to a rise in the GC content, unlike insertions which rather provoked a marked dip in the GC content. Together, indels rather lowered the GC content. However, the sum of substitutions and indels did not impact the overall GC content of *PpSat2* (Supplementary Figure S1).

**FIGURE 5:**
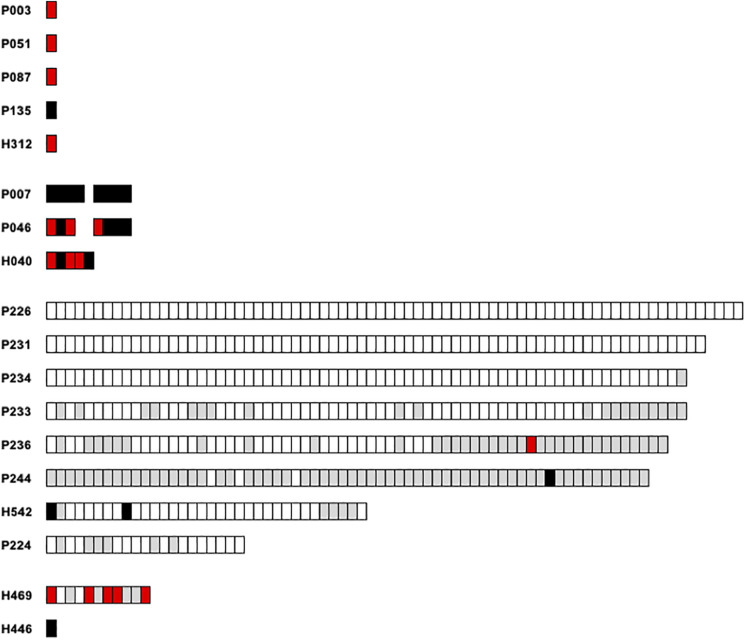
Graphic representation of the *PpSat1* repeats according to their deviation to the consensus sequence. Each rectangle represents a monomer. Repeats strictly identical to the consensus are indicated as white rectangles. Repeats containing at least one indel are colored in gray, repeats harboring only substitutions are represented by black rectangles, and repeats harboring both substitutions and indels are colored in red. Only entire repeats are presented.

We further intended to correlate the nucleotide variation of monomers of each satellite family to their relative location within the various arrays. Only entire repeats were retained for this analysis. Concerning *PpSat1*, two scaffolds (P226 and P231) were exclusively composed of the consensus sequence, which also constituted 67/68 repeats of a third scaffold (P234, Figure 5). Conversely, repeats from the scaffolds H446, H469, P236, and P244 largely diverged from the consensus, especially through deletions. As a consequence, these arrays consisted of slightly shorter repeats. Hence, 59 out of the 64 repeats identified in P244 and 36 out of the 68 repeats of P236 were shorter than the consensus sequence. In addition, all repeats located in low copy arrays and solo monomers were variants (Figure 5). Lastly, some scaffolds appear to have an intermediate organization, with a variable proportion of repeats identical to the consensus. Looking at the mutated repeats across the HC arrays revealed that they are preferentially located at the 5′ end and 3′ ends, while the central regions of the arrays were generally identical to the consensus (Figure 5). Noticeably, mutated repeats from HC arrays generally displayed deletions, while substitutions were predominantly found in LC arrays and solo repeats which all diverged from the consensus (Figure 5). Assuming that this rule is conserved among arrays, we anticipated that the repeats found in scaffolds H446 and H469 might belong to low copy arrays. Unlike what was observed in *PpSat1*, no particular hierarchical or structural organization could be characterized among *PpSat2* arrays.

**FIGURE 6:**
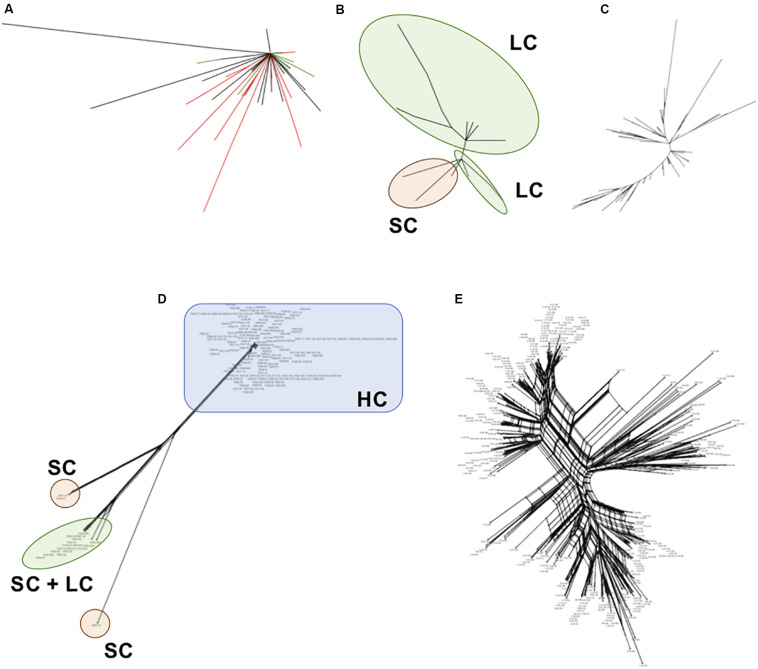
Phylogenetic relationships between representative repeats of *PpSat1* and *PpSat2*. **(A)** Analysis of the whole *PpSat1* dataset. Branches corresponding to repeats from HC arrays are indicated in black, those from LC arrays and solo repeats in red, and branches from unassigned repeats in green. **(B)** Analysis of LC arrays and solo repeats, excluding HC array-derived sequences. **(C)** Analysis of representative monomers from *PpSat2*. The trees were constructed using the Maximum Likelihood (ML) method based on the Tamura 3 model with a gamma rate of heterogeneity and 500 bootstrap replicates. **(D)** Phylogenetic network of *PpSat1* sequences. **(E)** Phylogenetic network of *PpSat2* sequences. (Phylogenetic networks were constructed using the SplitsTree software with the Neighbor-Net and uncorrected p-distance parameters. HC, high copy; LC, low copy; SC, single copy).

We inferred phylogenetic relationships among each satellite family, using the maximum likelihood (ML) method. The *PpSat1* repeats were mainly clustered according to their arrays of origin. Hence, most of sequences from the HC arrays were tightly clustered into a central location group, from which few sequences emerged, among which are the solo repeats, the monomers from the LC arrays, few HC repeats, and monomers located at ambiguous loci (Figure 6A). Focusing on the LC arrays and solo repeats revealed a further differentiation of the solo repeats which formed a distinct group with the 5′ distal repeats of the LC arrays (Figure 6B). By contrast, *PpSat2* sequences were poorly resolved into loose groups (Figure 6C), as expected from pairwise comparisons. To refine the relationships among repeats, we calculated a neighbor-net network of *PpSat1* and *PpSat2* monomers (Figures 6D,E). The SplitsTree supported a major clade within *PpSat1*, consisting of all HC arrays, while LC monomers and solo repeats were located on three distinct branches (Figure 6D). The topology of the tree suggested that the diversification of *PpSat1* proceeded from successive events rather than random mutations. On the opposite, we observed a very complex structure of interactions among *PpSat2* sequences, as illustrated in Figure 6E. Hence, despite an overall strong conservation among repetition units of each satellite family, analyses revealed two different evolutionary trajectories.

### *PpSat1* and *PpSat2* Have Complex and Distinct Genomic Locations

To better explore the possible function of the satellite DNA families, we undertook the characterization of sequences located at the vicinity of *PpSat* arrays. The scaffolds containing ≥64 copies of *PpSat1* were exclusively composed of repeats and could not be mapped to a particular region of the genome. They could not be assembled into longer scaffolds, because their size (20–25 kb) broadly corresponds to the average maximal length of the reads generated by the PacBio technology in our project (Rhoads and Au, 2015) and their extensive conservation hampers further assembly, so that we could not deduce the precise length of *PpSat1* arrays at the chromosomal level. *PpSat1* sequences shared several features with centromeric satellite families from a range of organisms, such as the average length, the overall base composition, and global organization. In addition, centromeric satellite DNA elements are generally present in extremely high abundance, and as *PpSat1* was the most abundant family identified in the *P. parasitica* genome, it was a good candidate for a centromeric satellite. Two partial HC arrays and the “ambiguous arrays” of *PpSat1* were located in the 3′ moiety of their respective scaffolds, so that we could define four “5” flanking’ regions likely unrelated to satellites (Table 2). Comparison of the 5′ flanking regions revealed a near perfect alignment over 3.16 kb between P224 and H542 and more generally allowed determining three blocks (designated as blocks A–C) that were conserved between two to four scaffolds (Supplementary Figure S2). Searching for the presence of repetitive sequences and potential protein-coding genes in the respective flanking regions revealed large fragments of known TEs scattered all along the four scaffolds, including both class I (Gypsy and Copia) and class II elements, among which are MuDR-derived sequences (Supplementary Table S1). No protein-coding region of significant length or bearing any structural or functional domain could be identified in these regions, with the exception of TEs. This result suggested that *PpSat1* copies organized into HC arrays are located in TE-rich, gene sparse regions, reinforcing the hypothesis that they might constitute centromeric components.

The three identified LC arrays of *PpSat1* were interrupted by 1213–1624-bp insertions (Table 2). We analyzed the 5′ and 3′ regions flanking the satellite sequences over a 25–35-kb length, as well as the intervening sequences. The scaffold H040 shared a >26-kb region highly homologous (98% identity) to the P046 sequence and a 1084-bp conserved region at the boundary of the *PpSat* arrays with the P007 sequence. Conservation between P007 and P046 were weaker, although noticeable (67% identity). Additional regions of weaker homology were identified among these alignments, indicating the repetitive nature of these regions. The three intervening sequences could be aligned over a 1083-bp length with an identity of 99%. This region, which was located immediately 5′ upstream the *PpSat1* sequences, was found to be similar in length and sequence to the conserved region identified among the 5′ flanking regions (Block A). So, the so-called intervening sequences were reevaluated as a possible general, 5′ upstream component of the *PpSat1* arrays. We thus aligned 5′ flanking regions of HC and LC repeats as well as intervening sequences and identified a ∼1,080-bp region that was highly conserved among all sequences that was associated with the *PpSat1* arrays (Supplementary Figure S3). A refined analysis of this 1,080-bp element showed that its 5′ end consisted in imperfect repetitions of the TTTAGGG motif, a typical feature of telomeric sequences, observed in plants and oomycetes, while mammals and most fungi rather display a (TTAGGG)_*n*_ sequence. Such motifs, found in a TE-rich region associated with satellite DNA, corresponded to interstitial telomeric regions, frequently observed in vertebrates and plants but not yet in oomycetes. The alignment revealed additional regions that were conserved among the various flanking regions, as a reflection of the numerous TE-derived sequences populating them (Supplementary Table S1). Investigation of the available 3′ flanking regions first revealed a 182-bp tandemly repeated sequence organized as a 40-unit array at the H040 locus and a 9-unit array flanking the P046 array, which was absent in the P007 locus. Extending the investigation revealed a ∼29.5-kb-long alignment with a 99% identity between H040 and P046 loci. The sequence located 3′ downstream the *PpSat1* repeats in the P007 scaffold did not share any homology with other 3′ flanking regions, although all of them mainly consisted of retrotransposon-derived sequences (Supplementary Table S1).

We explored the genomic environment of the 5 *PpSat1* solo repeats. Four of them were located 5′ upstream a (TTTAGGG)_44__–__52_ stretch corresponding to a telomeric end. Each solo repeat was flanked on its 5′ end by the 1080-bp region already identified in LC and HC arrays. In the case of the scaffold P051, this region was interrupted by a ∼4.75-kb Copia-derived element. The fifth solo repeat, located on scaffold 135, was not directly associated with a telomere, although we could identify telomeric DNA at ∼52 kb downstream the satellite sequence. As observed with the other repeats, it was flanked by the 1,080-bp block at its 5′ end. Exploring adjacent regions revealed significant regions of homology with sequences flanking the *PpSat1* arrays among which are a large proportion of TE relics, and no indication of protein-encoding genes (Supplementary Table S1). Additional searches in the *P. parasitica* genome did not reveal independent copies of the 1,080-bp block, which thus was considered as a component of the *PpSat1* machinery. We anticipated that this block should participate to the biogenesis of *PpSat1* and compared the two sequences, which turned out to display significant homologies, with a marked conservation in the 3′ moiety of *PpSat1* (Supplementary Figure S4). So, this homology would likely explain the constant association of these two sequences, which at least have a common origin. Another possibility is that one sequence originates from the other.

Performing the same analysis with the *PpSat2* elements revealed several features. Four *PpSat2* were interrupted by intervening sequences of 2,736–2,994 bp (Table 2). Yet, the resulting 5′ and 3′ arrays were arranged in a head-to-tail orientation, a situation prone to recombination. Flanking regions and intervening sequences from all scaffolds were submitted to a global alignment. As observed for *PpSat1*, several cases of high similarities were revealed among these regions that were scrutinized for TEs and protein-encoding genes. Compared to *PpSat1* flanking regions, they contained few TE fragments, but housed a significant proportion of protein-encoding genes, which spanned up to 81% of the total sequences flanking *PpSat2* (Supplementary Table S2). They could be assigned to a wide array of functional categories, including metabolism, signaling, effectors possibly involved in virulence, cytoskeleton organization, and transcription, as well as a large set of hypothetical proteins of unknown function, so that no bias could be observed in the nature and potential function of genes located in the vicinity of *PpSat2*. Nonetheless, this result indicated that *PpSat2* members were located in regions enriched in genes while *PpSat1* is rather located into gene sparse regions. This latter finding reinforced other observations pointing out the divergences between *PpSat1* and *PpSat2* and suggesting that they may fulfill distinct functions, if any.

### Conservation of *PpSat* Families Among Oomycetes and Putative Transcription in *P. parasitica*

A hallmark of satellites is their ability to evolve rapidly, so that the presence of *PpSat1* and *PpSat2* outside *P. parasitica* appeared unlikely. Yet, conservation of these sequences among *Phytophthora* spp., if not oomycetes, would suggest that they constitute ancient families that have been fixed, and are submitted to a strong selection pressure. We thus investigated oomycete genomes publicly available. Unfortunately, a majority of them were generated by short read sequencing technologies, so that SatDNA and other repetitive fractions escaped amplification prior to sequencing or were collapsed during the assembly process. Consequently, we generally failed to find *PpSat* analogs, except an incomplete copy of *PpSat1* in two *P. parasitica* strains (CJO1A1 and CJ05B6), and several hits in the genomes of two strains designated as *Phytophthora nicotianae* which were sequenced using the PacBio technology. This finding was expected, as both *P. nicotianae* and *P. parasitica* denominations correspond to the same species. Satellite repeats were identified on 31 and 33 contigs from the *P. nicotianae* genomes, respectively, and they were present as solo repeats or arranged in arrays of 2–57 copies. They were generally identical to the *PpSat1* consensus, as a reflection of probable concerted evolution among *P. parasitica*. We could define only one and two HC arrays in the two genomes, due to the relatively small length of the *P. nicotianae* contigs. Anyway, we could identify telomeric sequences composed of (TTTAGGG)_5__–__65_ stretches that were associated with *PpSat1* repeats in 14/31 contigs of one strain.

*PpSat1* matched with several genomic sequences from *P. infestans* and *Phytophthora cactorum*, and more surprisingly with three contigs from *Aphanomyces stellatus*, an oomycete taxonomically distant from *Phytophthora*. Of interest, *Aphanomyces* sequences were strictly identical to *PpSat1*, while homologs from *P. infestans* and *P. cactorum* were more divergent (Supplementary Figure S3). Unfortunately, the small length of the *Aphanomyces* contigs did not allow determining the length of the repeat and the complexity of arrays within this oomycete. We thus focused our comparison on the *Phytophthora* sequences. The *P. cactorum* contigs generally contained two tandem repeats of different length and relatively divergent, so that the precise length of the repetitive unit could not be determined. *P. parasitica* and *P. cactorum* sequences could be aligned over a 240-bp on the 5′ moiety of the monomer (Supplementary Figure S5A). The *P. infestans* contigs displayed until 27 repeats in a single contig. The consensus repeat was 337 bp long, with a GC content of 34% and which displayed 73.94% identity to *PpSat1* (Supplementary Figure S5B). Blastn searches against the *P. parasitica* genome did not reveal a better match than *PpSat1*. As expected, we found relics of the 1,080-bp block flanking *PpSat1* analogs in all species, including *A. stellatus*, confirming the common origin of this sequence and *PpSat1*. It also confirmed that sequences retrieved in Blastn searches were actually homologs of *PpSat1*. In contrast, *PpSat2* homologs were found only within *P. nicotianae* contigs, where they constituted arrays of 19–59 repeats. Single hits were observed in various *P. parasitica* genomes resulting from short read sequencing projects, indicating that they probably were collapsed during assembly and simply missed in the current genome assemblies.

We then investigated the transcriptional profiles of *PpSat1* and *PpSat2* and the 1080-bp-block in *P. parasitica*. To this aim, we performed Blastn searches against the various RNA-Seq libraries available at GenBank. RNA-Seq reads were generated from various pre-infection stages of *P. parasitica*, as well as two different infected plants. We also mined two libraries enriched in small RNAs. As a rule, the three sequences were represented by a paucity of reads in the various libraries corresponding to free-swimming zoospores, germinated cysts, sporulating hyphae, and vegetative mycelia, as well as lupine or Arabidopsis roots collected at various steps of infection (Supplementary Table S3). As a control, we evaluated the representation in these libraries of *WS41*, a *P. parasitica* gene usually considered as constitutively expressed. The situation was quite different when exploring libraries enriched in sRNA. Hence, the three sequences were in the mycelium library enriched in small RNAs, as well as in the small RNA library prepared from later stages of Arabidopsis infection (Supplementary Table S3). Reads covered the whole sequence of *PpSat1*. In contrast, sRNAs were identified throughout the entire *PpSat2* region at the exception of a ∼45-bp fragment located at the 5′ end. Lastly, the 5′ and 3′ distal regions of the 1,080-bp block were largely covered more by reads than its central region. Not only did the three sequences differ by the extent of their coverage by sRNA, but also the size of their corresponding sRNAs varied in a sequence-specific manner. Hence, the sRNAs were 28–38 nucleotides long for *PpSat1*, 28–33 nucleotides for the 1,080-bp block, and 28–29 nucleotides for *PpSat2*. No significant hits were found for *WS41* in the sRNA-enriched libraries (Supplementary Table S3), indicating that the reads matching the satellite families correspond to genuine sRNAs and do not correspond to degraded mRNA molecules. There was no bias in sRNA orientation, as both sense and antisense reads were identified in quite a similar amount whatever the satellite sequence that was analyzed.

## Discussion

### Updated Genome Assembly Uncovers Two Satellite DNA Families in *P. parasitica*

We present here two classes of tandemly repeated DNA which may be considered as the first satellite DNA families from oomycetes to be described. This class of repetitive DNA has been understudied until now within this group of lower organisms despite its potential importance to our understanding of genome architecture and evolution of most eukaryotes. This may be explained by the fact that satellite DNA elements were generally not identified using short read (e.g., Illumina) sequencing and *de novo* assembly, because aligning and assembling highly conserved sequences constituted an important computational challenge (Treangen and Salzberg, 2012; Lower et al., 2018). This is illustrated by *PpSat1* and *PpSat2* whose monomers are longer than the Illumina average read length and that were probably collapsed during assembly. Another problem encountered is that short-read library preparation steps introduce a bias against amplification of GC-rich sequences, like *PpSat2*, which may result in their under-representation (Lower et al., 2018). With a N50 of ∼15 kb, the updated assembly of *P. parasitica* PPINRA-310, based on the use of the PacBio sequencing technique, allowed filling approximately 30 Mb of gaps and uncovered two satellite DNA families. Yet, we were unable to precisely determine the extent of the various arrays that constitute these families. Further investigations on the satellite complement of the *P. parasitica* genome would require specific computational pipelines rather developed on unassembled reads on a range of organisms (Garrido-Ramos, 2017). Alternatively, the generation and further assembly of ultra-long reads would provide knowledge on the satellite catalog of this pathogen. Yet, the aim of the present sequencing project was essentially filling gaps in the whole genome and not an exhaustive repertoire of repetitive sequences.

The repeat length and the AT content of *PpSat1* are similar to those found in satellites from insects (Ugarković et al., 1996) and plants (Li et al., 2019) and more generally to centromeric satellites whose canonical size of 170 or 340 bp has been proposed to correspond to the size of one or two nucleosome units (Grellet et al., 1986; Henikoff et al., 2001; Heslop-Harrison and Schwarzacher, 2013). Satellite DNA families are often AT rich (Plohl et al., 2008), and homopolymeric stretches may be identified across the sequence (Ugarković et al., 1996). Such features have been proposed to induce DNA curvature and to ease the packing of DNA and proteins in the heterochromatin, providing satellite DNA with a crucial function (Ugarkovic, 2005; Escudeiro et al., 2019). Such stretches are present in *PpSat1*, which therefore potentially contributes to the DNA bendability. GC-rich families were also detected (Petrovic et al., 2009). Therefore, *PpSat2* was also proposed to form a satellite family, with a >57% GC content. Despite their respective biased GC contents, *PpSat1* and *PpSat2* have the potential to adopt complex secondary structures. Such structures could be under selective constraint, due to the overall sequence conservation among each family, and possibly participate to interactions with other nucleic acid molecules or DNA-binding proteins, as shown for satellite repeats in a number of cases (Ugarkovic, 2005; McNulty and Sullivan, 2018).

### Distinct Trajectories Govern the Dynamics of *PpSat1* and *PpSat2*

*PpSat1* and *PpSat2* diverge by several criteria, among which is their diversity. Hence, *PpSat1* repeats broadly fit into two categories: a prevalent sequence, which represents 66% of the dataset, and a myriad of unique sequences differing from the primary monomer by slight differences, which overwhelmingly consist of deletions. Whether the major monomer is the founder sequence is highly probable, as it was found in all HC arrays, while all LC arrays and solo repeats constituted variants. This potential founder repeat is distributed neither equally nor randomly across HC arrays. Hence, some arrays are composed of this single sequence, while other arrays mainly contain variants. Using the PacBio technique provides reads spanning tens of contiguous monomers, so that the observed situation does not result from artifacts occurring during the assembly process, but rather corresponds to the actual organization of *PpSat1*. Whether the arrays identified in the present work are contiguous on the genome or represent parts of longer arrays. It is also impossible to determine whether the divergent HC arrays are specific to distinct chromosomes, as already observed (Roizes et al., 1980; Gong et al., 2012). Consequently, we cannot estimate the number of chromosomes in *P. parasitica* via the characterization of centromeric sequences. It has been shown that plant and animal chromosomes may lack tandem repeats, and it may occur in oomycetes as well (Nasuda et al., 2005). The situation observed in *PpSat2* is totally different, as no major sequence could be evidenced, so that we face a bloom of variant sequences and it is impossible to infer a potential founder sequence. Yet, a deeper analysis of mutations within *PpSat2* provided unexpected clues on the evolution of this sequence. Hence, we showed that substitutions accumulated in few GC “poor” regions and contributed to a GC enrichment, while indels globally provoked a dip of the GC content, more generally located in regions otherwise exhibiting a GC count higher than the average content of the entire monomer. Finally, there was no global change in the GC content of *PpSat2*. This observation might reflect a trend to maintain a global stability in the GC content of *PpSat2*. So, we can suppose that although no clear founder sequence was characterized among *PpSat2* monomers, selection pressure acts, through targeted substitutions and indels at preferred sites, to maintain a given homeostasis of the GC content and limit the impact of possible local changes in base composition and consequently on the potential secondary structure of *PpSat2*, which might govern its function. It also indicates that distinct mechanisms drive *PpSat1* and *PpSat2* evolution, the former mainly evolving via indels, the latter via a strict conservation of its base composition.

The coexistence of a major sequence and minor variants in *PpSat1* and the overall high conservation is likely the result of a molecular drive, an important force commonly proposed to drive the evolution of satellite DNA (Dover et al., 1982) leading to concerted evolution of the whole family (Waye and Willard, 1989). Following this model, sequence homogenization would occur through various mechanisms such as unequal crossing-over, which have been predicted to be less efficient in bordering regions, so that distal arrays would be more divergent that those located centrally within the array (Plohl et al., 2008). This situation is observed in several scaffolds bearing *PpSat1* sequences, in accordance with the concerted evolution theory. The accumulation of mutations among *PpSat2* members would suggest that these sequences are resistant to concerted evolution, although a global analysis revealed that mutations accumulated in few locations and at high frequency, leading to the fixation of sequence variants, another expression of sequence homogenization. A possibility is that *PpSat2*, located in gene-rich regions, is not prone to global chromosomal rearrangements, while the genomic environment of *PpSat1*, mainly composed of TEs, favors reshuffling and homogenization and the spreading of this satellite family toward different genomic regions such as telomeres.

The occurrence of *PpSat1* either as solo repeats in the vicinity of telomeres or in arrays containing tens of monomers may shine a light on the possible evolution of this satellite family and apparently fit the model established by MacGregor and Sessions from analysis of newt satellites (MacGregor and Sessions, 1986). In this model, satellite DNA tends to accumulate at discrete locations like centromeres through tandem duplication and then is subject to chromosomal rearrangements, which frequently occur in these regions. These rearrangements induce a progressive dispersal of satellite DNA from the centromeres to pericentric regions, leading to a further dispersal to the telomeres, where the sequences degenerate and ultimately become undetectable following accumulation of mutations (MacGregor and Sessions, 1986). According to this model, HC arrays, LC arrays, and telomeric solo repeats might correspond to three distinct steps of the dispersal pattern of *PpSat1* across the genome, illustrated by their phylogenetic relationships and their relative extent of deviation toward the consensus sequence, which is highly likely representative of the founder sequence. Fitting this model would also reinforce the hypothesis of a centromeric location for HC arrays.

### Searching a Function for Satellite DNA Families From Comparative and Transcriptome Analyses

Sequence homologs to *PpSat1* were found in at least two *Phytophthora* species. It has to be noted that the *P. cactorum* repeats were relatively divergent and present in tandem of unequal length. In contrast, the *P. infestans* homolog retained the high copy organization and monomer length and displayed ∼75% identity, in frame with the phylogenetic affinity between *P. infestans* and *P. parasitica* (Yang et al., 2017). Maintaining the strict conservation of length, rather than sequence, suggests a structural role for these satellites. More surprisingly, truncated copies of *PpSat1* are strictly conserved in *A. stellatus*, a distant oomycete opportunistic pathogen (Gaulin et al., 2018). In the absence of longer scaffolds, it was impossible to evaluate properly the length of the conserved monomers and their abundance. A specific mining of oomycete genomes would indicate whether *PpSat1* was present in the precursor of *Phytophthora*, and maybe in other oomycete lineages, and whether it is also present in tandemly repeat copies. Conversely, *PpSat2* appears to be specific to *P. parasitica*. It may imply that this satellite is of more recent origin than *PpSat1*, a hypothesis illustrated by the lack of major repeat and the high mutation rate. Alternatively, it may correspond to an old satellite family which accumulated unfixed mutations, so that it could not be detected in other *Phytophthora* species. Comparative analysis of repeats from several *P. parasitica* strains would reveal insights into the evolution of this family.

*PpSat* 1 and *PpSat2* are harbored by distinct scaffolds. In addition, *PpSat1* arrays are generally flanked by TE islands while *PpSat2* arrays are located close to gene-rich regions. The base composition of each family may participate to these distinct locations. Hence, an AT-rich composition is a frequent feature of centromeric repeats. With an increased GC content, *PpSat2* may be more suited to adapt to the overall GC content of the *P. parasitica* genome, at least of the gene-rich regions, as a strategy to persist within this environment. The observation that the GC percentage varies across the monomer consensus, resulting in local rises and dips compared to the mean content, but remains globally unchanged through accumulation of non-random substitutions and indels that maintain a global “GC homeostasis,” strikingly supports the hypothesis that the base composition and consequently the potential secondary structure of *PpSat2* are submitted to a strong selection pressure.

The accumulation of differences may suggest that, if functional, *PpSat1* and *PpSat2* may participate to different processes. Yet, mining RNA-Seq data revealed that they display highly similar transcriptional patterns, indicative of an apparent co-expression, if not co-regulation. Hence, the two families, as well as the PpSat1-associated 1,080-bp block, present a very faint expression level in pre-infection structures, as deduced from a handful of reads in RNA-Seq experiments. Moreover, these sequences appear to be repressed during plant infection. Yet, these three sequences are clearly identified in RNA-Seq data from libraries enriched in small RNAs, although at different levels. *PpSat1* is by far the most expressed sequence, while *PpSat2* is largely less represented in the libraries. The 1,080-bp block displays an intermediate situation. This observation reinforces the idea that this fragment, which is always associated with *PpSat1* and shares with this latter sequence significant homology, may constitute a component of the *PpSat1* machinery. The observation that the three sequences are mainly transcribed in the form of sRNA suggests that epigenetic mechanisms regulate their expression.

The importance of small RNA-based silencing and epigenetics in the biology and pathogenicity of *Phytophthora* has emerged with the evidence of silencing events (van West et al., 2008), the identification of the silencing machinery, and the characterization of sRNAs in various species (Vetukuri et al., 2012; Fahlgren et al., 2013; Åsman et al., 2016; Bollmann et al., 2016, 2018; Wang et al., 2016; Jia et al., 2017). These studies revealed that *Phytophthora* possess several small RNA populations diverging by their size (Vetukuri et al., 2012; Fahlgren et al., 2013). Among them, two main classes were centered on 21 and 25–26 nucleotides (nt), which may fulfill distinct roles in the silencing mechanisms (Åsman et al., 2016; Jia et al., 2017). The two satellite families and the associated 1080-bp block differed by the mean length of matching sRNAs, their relative abundance, and their location across the sequence. The reads identified in the sRNA libraries matched *PpSat1* on longer regions (28–38 nt) and spanned its entire region with an abundant coverage. Matches with the associated 1080-bp block were shorter molecules (28–33 nt) and rather accumulated at both 5′ and 3′ regions, while the central region displayed a lower coverage. The global number of corresponding reads was 10-fold less abundant than in the case of *PpSat1*. Lastly, shorter, homogeneous (28–29 nt) regions of homology were identified with *PpSat2*. They were largely (∼50-fold) less abundant than *PpSat1* sRNA and mapped the entire region at the exception of the 45-bp 5′ end. Interestingly, this region largely exceeded the average GC content, locally reaching 70% (Figure 2). Whether the level of expression of each sequence is correlated with their relative copy number or to their relative percentage of the genome content is unknown and would merit further explorations. These studies would also allow determining whether *PpSat1* and *PpSat2* are targeted by distinct classes of sRNA varying in size as supposed from the observed length of the matches in blast analyses. More generally, it remains to know whether sRNAs targeting satellite DNA constitute a distinct class, in addition to the main 21-nt and 25–26-nt classes identified in *Phytophthora*, including *P. parasitica* (Jia et al., 2017).

The differences in the relative expression of the satellite families, as deduced from the number of reads in the mycelium sRNA libraries, were similar to those scored in the sRNA libraries from *A. thaliana* roots infected with *P. parasitica*. sRNAs matching the satellites were observed only at 24 h post inoculation. This likely reflects the accumulation of *Phytophthora* biomass during plant infection, so that we may suppose that the expression of *PpSat1* and *PpSat2* in the form of sRNA occurs all along the *Phytophthora* life cycle. This finding is not totally unexpected. Hence, long considered as junk DNA, satellite DNA has been shown to be transcriptionally active in a number of organisms, especially in the form of siRNA (Ugarkovic, 2005; Biscotti et al., 2015a). This activity would participate to centromere biogenesis, kinetochore formation through recruitment of centromere proteins, and heterochromatin formation at both centromeric and telomeric regions (Biscotti et al., 2015b; McNulty and Sullivan, 2018). Whether this observed expression actually corresponds to a constitutive repression through a silencing mechanism has been poorly documented. Yet, satellite repression is required for maintaining genome stability (Zeller and Gasser, 2017). Satellite-derived sRNAs may be induced upon stress (Pezer and Ugarkovic, 2012; Brajković et al., 2018). In addition, alpha satellite derepression was linked to DNA damage, mitotic errors, and genome instability (McNulty and Sullivan, 2018). So, we can hypothesize that *PpSat1* and *PpSat2* are repressed by siRNA-mediated silencing under normal conditions, and further studies on the *Phytophthora* stress response would provide invaluable information on the PpSat biology. *PpSat1* possesses a range of features characteristics of centromeric SatDNAs and therefore is a good candidate to ensure such functions. The role of *PpSat2* is totally unknown to date. A refined exploration of the distribution and evolutionary dynamics of this family among *P. parasitica* would accelerate the elucidation of the role of the two present satellite families in the biology and pathogenicity of this important pathogen.

## Data Availability Statement

The datasets generated for this study can be found in GenBank, accessions MT439330 and MT303065.

## Author Contributions

FP designed the study, mined and interpreted the data, performed the sequence analyses, and wrote the manuscript. CR performed genome assembly. MR performed small RNA analyses and interpreted the results. M-LK conducted and maintained *Phytophthora* cultures, extracted genomic DNA for library preparation, and analyzed sequences. All authors reviewed and approved the final manuscript.

## Conflict of Interest

The authors declare that the research was conducted in the absence of any commercial or financial relationships that could be construed as a potential conflict of interest.

## Footnotes

1 https://en.vectorbuilder.com/tool/gc-content-calculator.html

2 http://rna.tbi.univie.ac.at

## Supplementary Material

FIGURE S1 impact of mutations on the GC content of *PpSat2*. Presented is the relative proportion of mutations (substitutions, insertions, and deletions) that induce a rise (higher GC) or a dip (lower GC) in the average GC content of *PpSat2*. Mutations were examined in separated analyses. Observed substitutions leading to a higher GC content were AC, AG, and TG. Substitutions toward a lower GC content were CA, CT, GA, and GT. Neutral substitutions were AT, CG and GC.

FIGURE S2 Schematic representation of the upstream regions flanking HC *PpSat1* arrays.

FIGURE S3 Partial nucleotide alignment of regions located 5′ upstream *PpSat1* repeats from HC and LC arrays, focused on the 1,080-bp conserved element. The motifs corresponding to interstitial telomeric regions are underlined. Int: sequences initially identified as interrupting the LC arrays. Conserved nucleotides are indicated by *.

FIGURE S4 Alignment of *PpSat1* and the 1,080-bp block.

FIGURE S5 Alignment of *PpSat1* with homologs from *P. cactorum* **(A)** and *P. infestans* **(B)**.

TABLE S1 Simplified description of Transposable Element (TE)-derived sequences flanking the various *PpSat1* arrays. TEs were searched at RepBase using the Censor tool (see section “Materials and Methods”).

TABLE S2 Potential protein-encoding genes associated with *PpSat2* arrays. Indicated are the extent of the ORF and its location on the flanking sequence, the gene model, its length in bp, the orientation of the ORF, and a short description. Below the description of each flanking region are indicated the total length of coding regions and the relative proportion of coding space. Putative transposable elements (indicated in bold, italic characters) were removed from the calculation of the coding space.

TABLE S3 Relative expression of *PpSat1*, *PpSat2* and the 1,080bp-block estimated as the total number of reads matching each sequence. *WS41* was used as internal constitutive control (see section “Results”).
