# Supplementary figures and images for: Characterization of Two Satellite DNA Families in the Genome of the Oomycete Plant Pathogen Phytophthora parasitica
Source: Front Genet. 2020 Jun 5;11:557. doi: 10.3389/fgene.2020.00557 (PMC7290008; doi:10.3389/fgene.2020.00557)

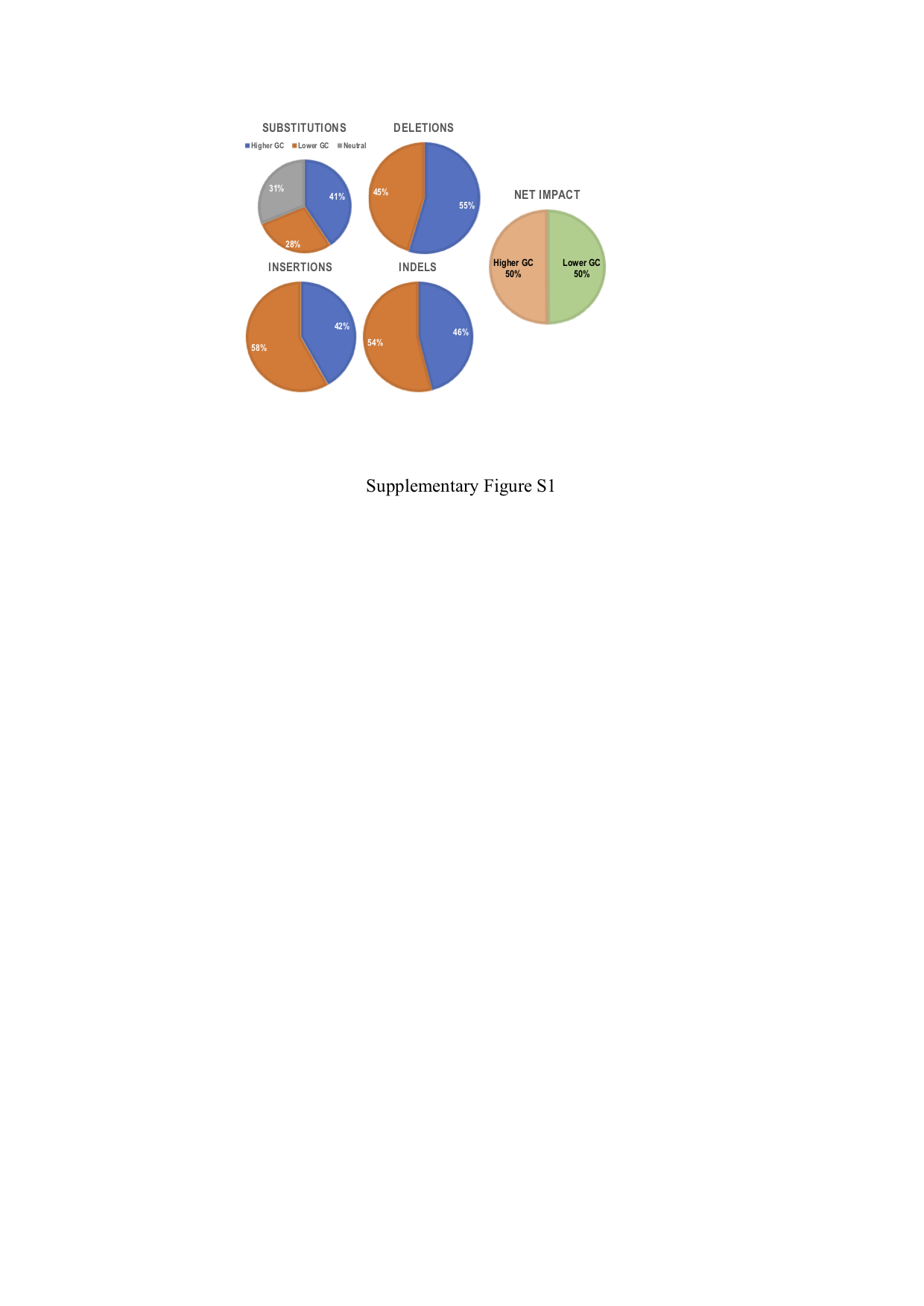

Supplement: FIGURE S1 — impact of mutations on the GC content of PpSat2. Presented is the relative proportion of mutations (substitutions, insertions, and deletions) that induce a rise (higher GC) or a dip (lower GC) in the average GC content of PpSat2. Mutations were examined in separated analyses. Observed substitutions leading to a higher GC content were AC, AG, and TG. Substitutions toward a lower GC content were CA, CT, GA, and GT. Neutral substitutions were AT, CG and GC. [file Image_1.TIFF]

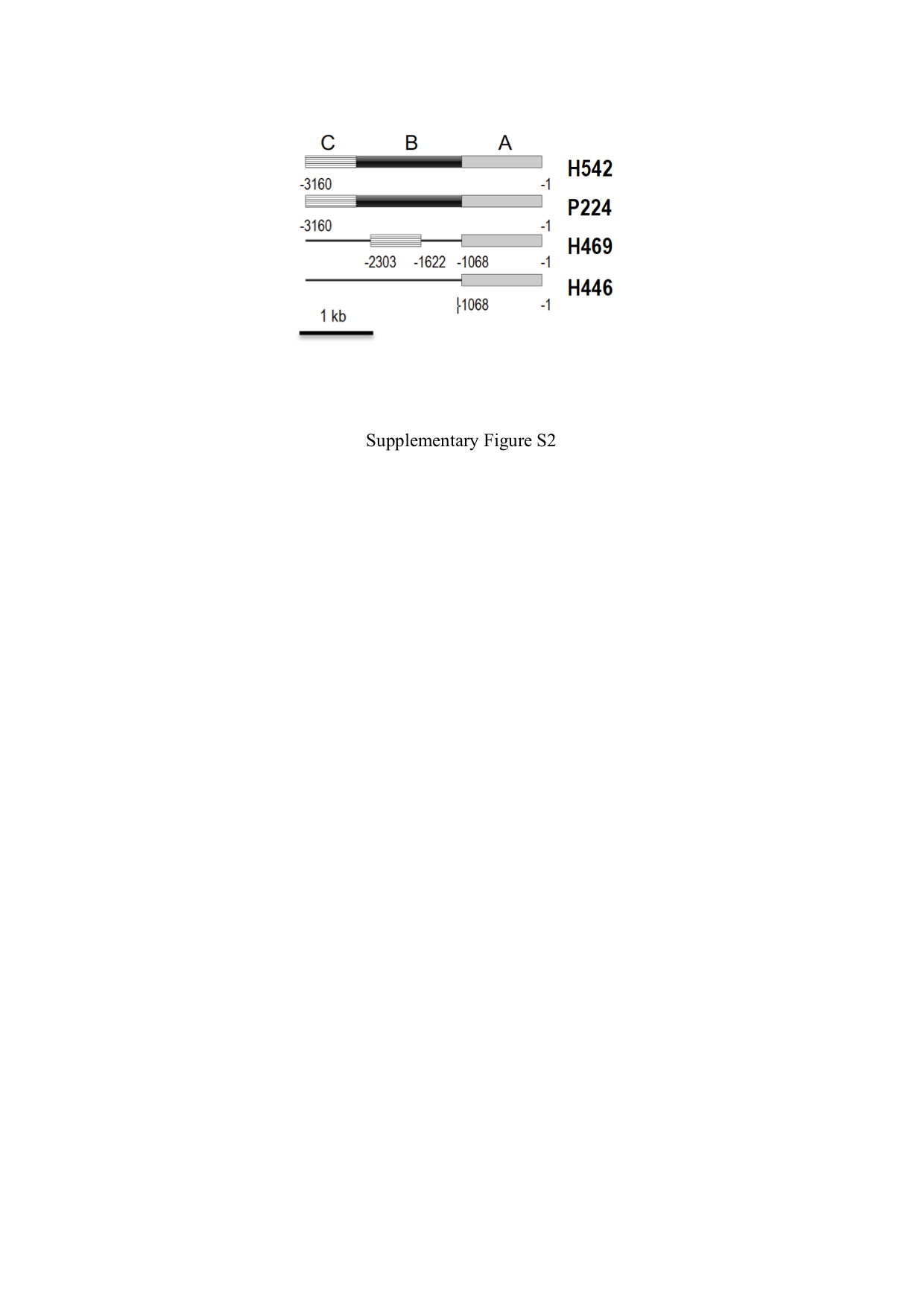

Supplement: FIGURE S2 — Schematic representation of the upstream regions flanking HC PpSat1 arrays. [file Image_2.TIFF]

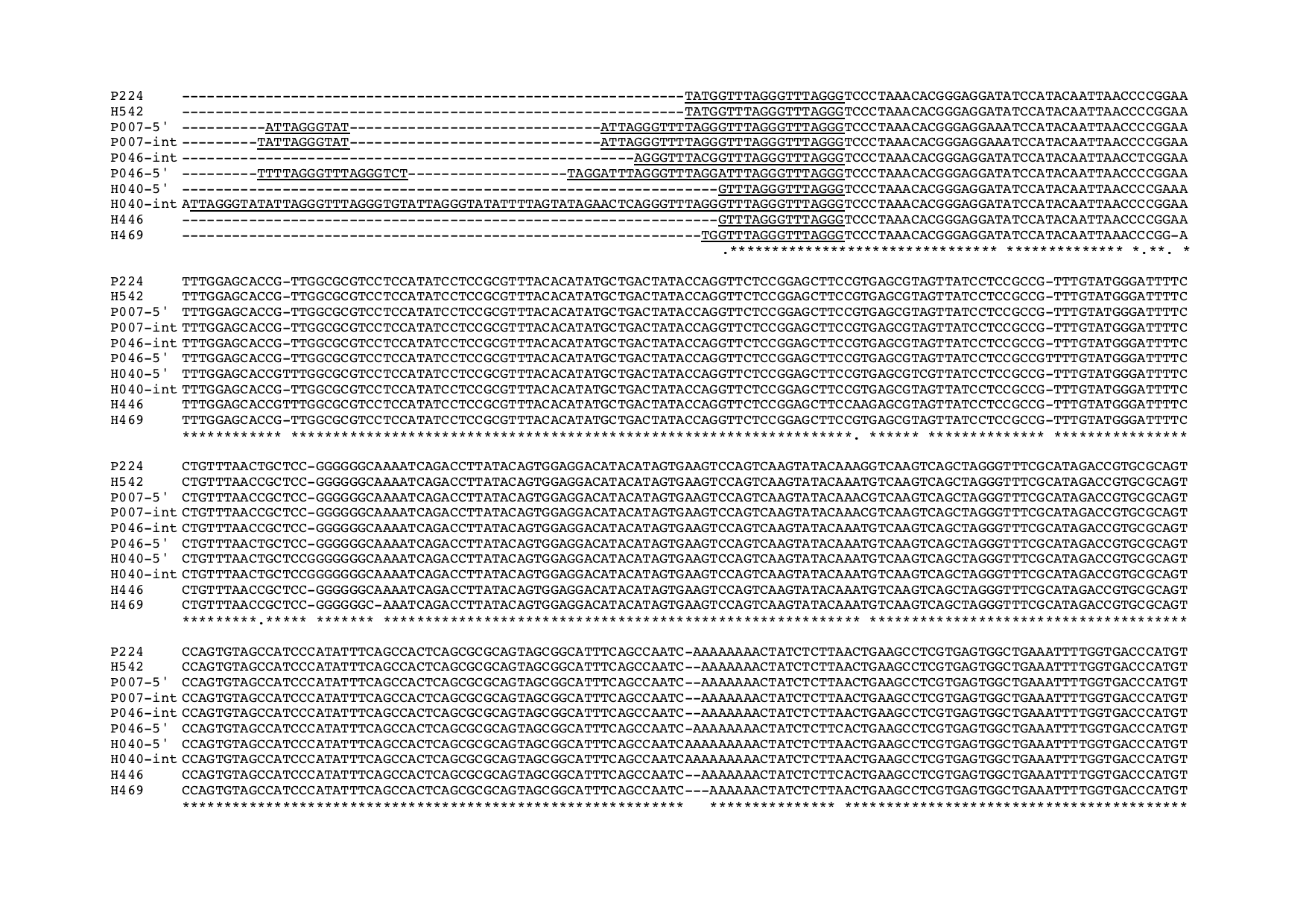

Supplement: FIGURE S3 — Partial nucleotide alignment of regions located 5′ upstream PpSat1 repeats from HC and LC arrays, focused on the 1,080-bp conserved element. The motifs corresponding to interstitial telomeric regions are underlined. Int: sequences initially identified as interrupting the LC arrays. Conserved nucleotides are indicated by *. [file Image_3.TIFF]

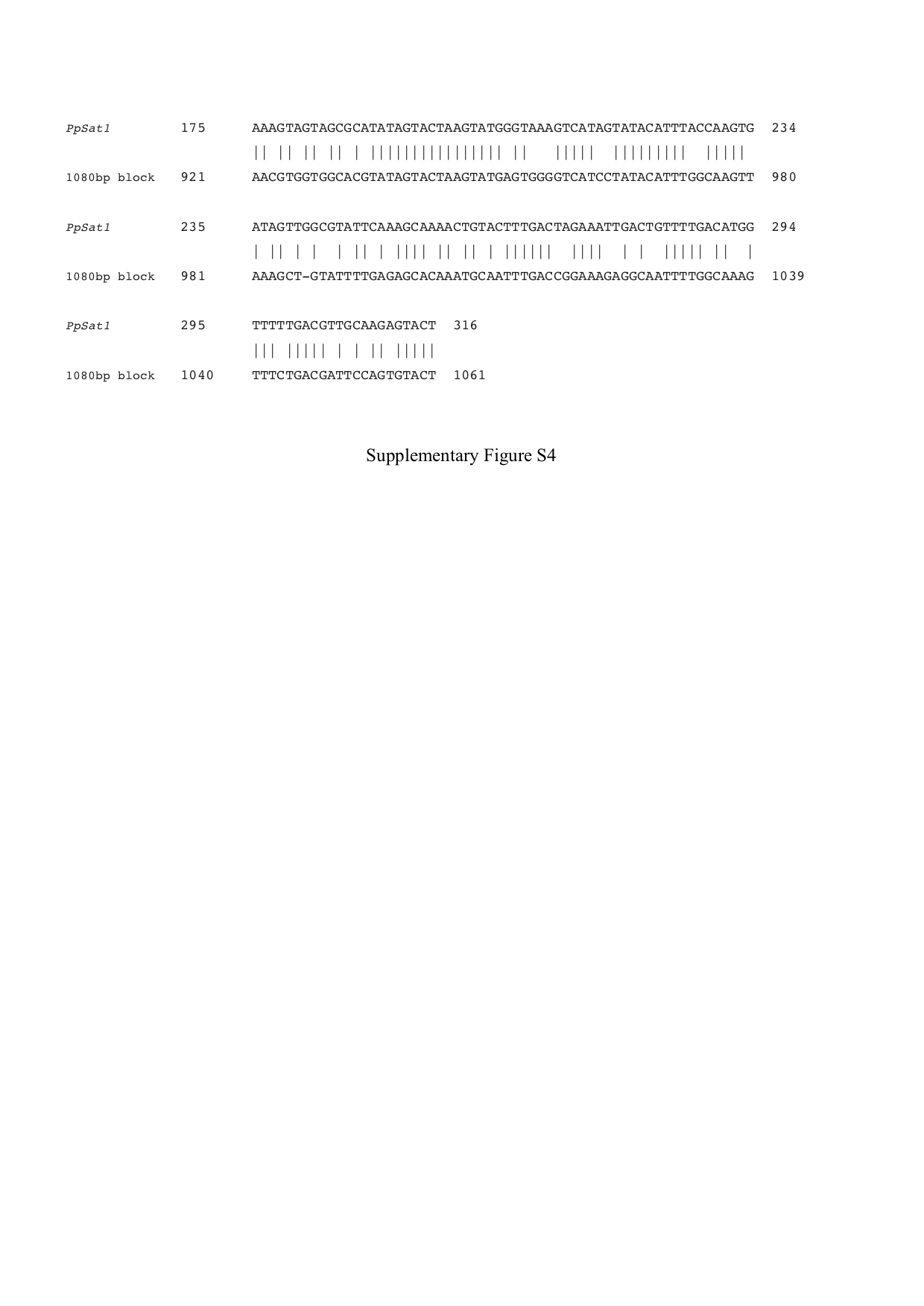

Supplement: FIGURE S4 — Alignment of PpSat1 and the 1,080-bp block. [file Image_4.TIFF]

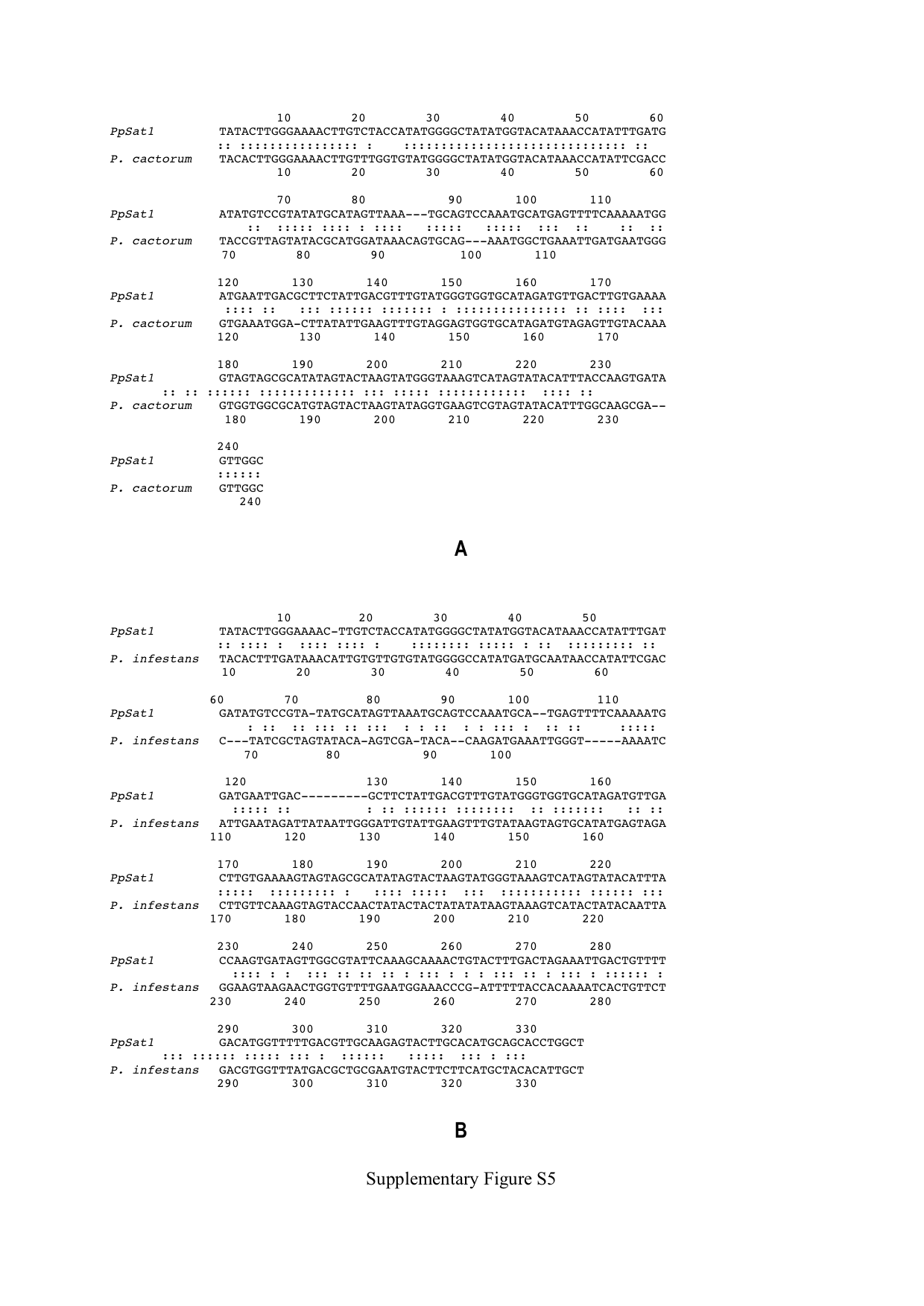

Supplement: FIGURE S5 — Alignment of PpSat1 with homologs from P. cactorum (A) and P. infestans (B). [file Image_5.TIFF]
